# The nightscape of the Arctic winter shapes the diving behavior of a marine predator

**DOI:** 10.1038/s41598-024-53953-w

**Published:** 2024-02-16

**Authors:** Philippine Chambault, Jonas Teilmann, Outi Tervo, Mikkel Holger S. Sinding, Mads Peter Heide-Jørgensen

**Affiliations:** 1 Greenland Institute of Natural Resources, Strandgade 91, 2, 1401 Copenhagen, Denmark; 2 Department of Ecology and Evolutionary Biology, The University of California, 1156 High Street, Santa Cruz, CA 95064 USA; 3 Marine Mammal Research, Department of Ecoscience, Aarhus University, Frederiksborgvej 399, 4000 Roskilde, Denmark; 4 Section for Computational and RNA Biology, Department of Biology, University of Copenhagen, 1350 Copenhagen, Denmark

**Keywords:** Cetacean, Foraging, Diving behavior, *Phocoena phocoena*, Daylength, Greenland, Ecology, Behavioural ecology

## Abstract

Predator–prey interactions in marine ecosystems are dynamically influenced by light, as demonstrated by diel vertical migrations of low-trophic level organisms. At high latitudes, the long winter nights can provide foraging opportunities for marine predators targeting vertically migrating prey closer to the surface at night. However, there is limited documentation of such diel patterns in marine predators under extreme light regimes. To address this, we recorded the diving behavior of 17 harbour porpoises just south of the Arctic circle in West Greenland, from summer to winter. Unlike classical diel vertical migration, the porpoises dove 24–37% deeper at night and the frequency of deep dives (> 100 m) increased tenfold as they entered the darkest months. The daily mean depth was negatively correlated with daylength, suggesting an increased diving activity when approaching the polar night. Our findings suggest a light-mediated strategy in which harbour porpoises would either target (i) benthic prey, (ii) pelagic prey migrating seasonally towards the seafloor, or (iii) vertically migrating prey that may be otherwise inaccessible in deeper waters at night, therefore maximizing feeding activity during extended periods of darkness. Extreme light regimes observed at high latitudes are therefore critical in structuring pelagic communities and food webs.

## Introduction

Natural light cycles play a crucial role in shaping the behavior of most animals on earth, with profound implications for ecological interactions and fitness consequences within animal communities^1^. While some species exhibit diurnal, nocturnal or crepuscular activity patterns, others adopt more complex diel patterns known as cathemeral activity, to partition their behaviors between vital activities such as predator avoidance, thermoregulation, and foraging^2^. This time partitioning plays a critical role in structuring terrestrial and marine food webs by mediating ecological interactions.

The three-dimensional nature of the ocean gives rise to dynamically structured marine ecosystems where predator–prey interactions are mediated by light and center around foraging optimization and predator avoidance^3^. This can be achieved through diel vertical migrations (DVM) whereby low-trophic level organisms migrate vertically in response to changes in light, providing foraging opportunities for predators^4^. By representing the largest animal migration in terms of biomass, DVM occurs in a wide range of both freshwater and marine zooplankton taxa, that is subsequently followed by vertically migrating predators from cephalopods and fish to the largest marine mammals^5^. However, in high latitude regions, DVM of zooplankton was previously expected to cease in response to extreme light regimes (midnight sun: 24 h of irradiance vs. polar night: 24 h of darkness)^6^. Despite such extreme light cycles, growing evidence indicates that DVM behavior of zooplankton may continue throughout the Arctic winter^7^, potentially making the polar night a system of high biological activity^8^.

Reverse diel vertical migrations (deeper nocturnal dives) have however been observed in some diving predators such as elasmobranchs^9,10^, seals^11^ and toothed whales^12,13^. While the reasons for reverse migration are still unclear, one hypothesis is that vertically migrating prey can be out of reach in deep layers during the day while being more accessible in upper layers at night^14^. This suggests that extended periods of darkness in winter may provide foraging opportunities for marine predators^8,15^ that access prey located closer to the surface while reducing energy expenditure. Diel variations in diving behavior may serve as a mechanism linking optimal foraging strategies with physiological processes. The ascent of prey at night shortens the predator's transit time, thereby facilitating the predator's ability to operate within its physiological constraints while optimizing energy acquisition.

Among the Arctic predators, the harbour porpoise (*Phocoena phocoena*) in Greenland inhabits one of the most northerly part of the species range in the sub-Arctic and Arctic waters, and is most abundant in coastal temperate and boreal waters of the Northern hemisphere^16^. This species experiences contrasting light regimes throughout its range, and is known to be more active at night^17–23^. Harbour porpoises in Greenland also exhibit a unique behavior by using both coastal shallow waters and deep offshore waters (up to 1000 m^24^), potentially feeding on prey distributed in the mesopelagic layer^25^ (> 200 m). Following the vertical migration of its prey, we expect the harbour porpoise in Greenland to perform deeper diurnal dives compared to nocturnal dives, responding to the reduction of daylength by increasing its diving activity (*i.e.,* dive depth and dive frequency). Although the diel pattern in the diving behavior of marine predators has been extensively studied in areas with relatively stable light regimes at lower latitudes, little is known about the effect of changing light on the vertical behavior of marine predators in Arctic ecosystems^26^ where light conditions vary greatly, ranging from the midnight sun to the polar night.

Given the occurrence of the harbour porpoise in sub-Arctic and Arctic waters^27^, it serves as an ideal study model to examine how the extreme cyclicity of light regime drives the diving behavior of this marine predator. To explore this, harbour porpoises were instrumented with Argos satellite linked dive recorders in West Greenland to record their at-sea distribution and diving behavior over six months, spanning the extreme light regimes from summer to winter. Dive data were first examined in relation to sunrise and sunset to identify potential diel and seasonal patterns. Subsequently, we investigated the relationship between diving behavior and daylength, to better understand how extreme light cycles at high latitudes may shape the behavior of a marine predator.

## Results

### Extreme light regimes experienced by harbour porpoises

**Figure 1:**
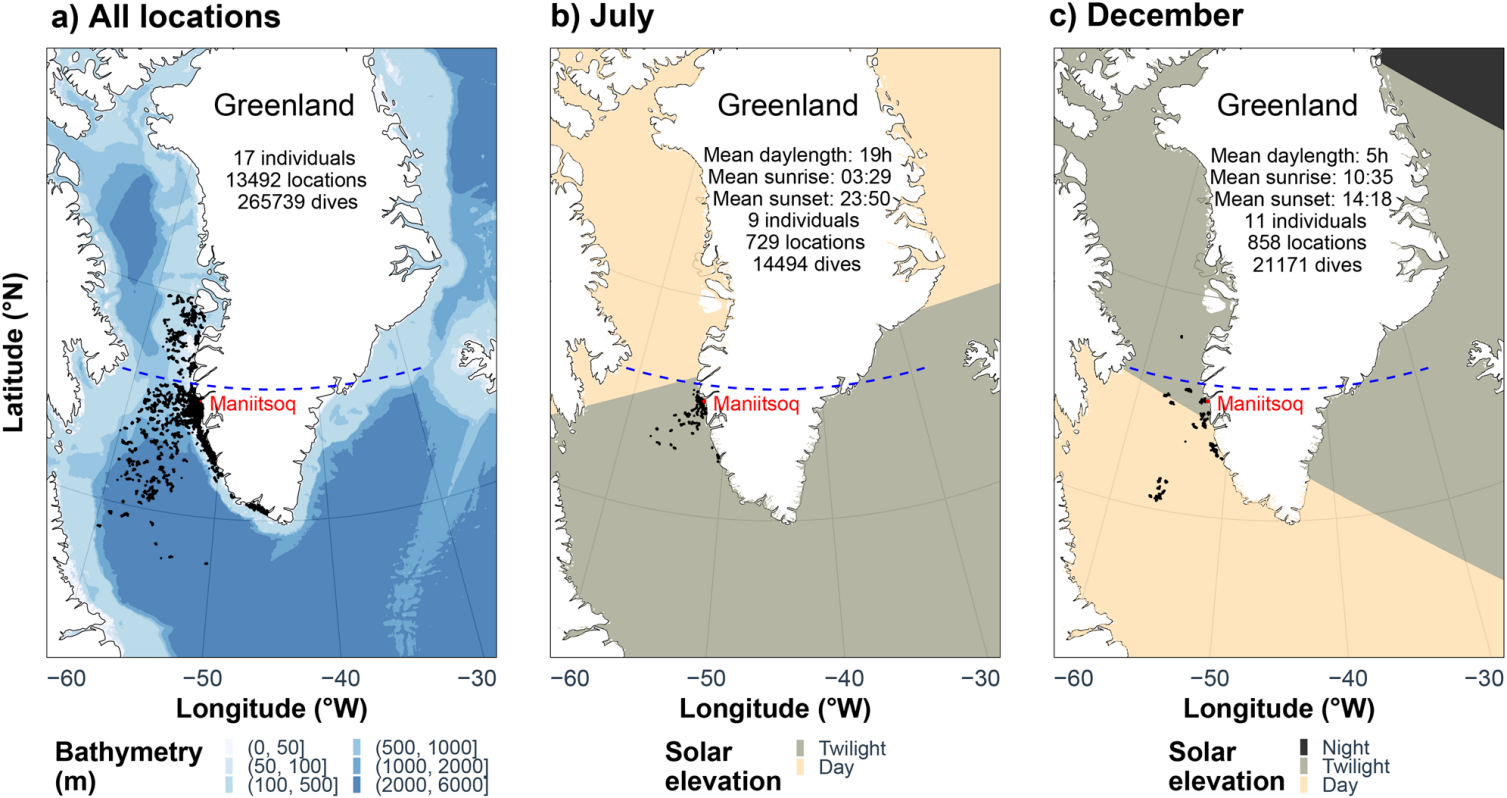
Maps of the study area showing the (**a**) bathymetry and the contrasted light cycles in (**b**) July and (**c**) December with all the associated locations recorded from the 17 satellite tracked harbour porpoises (black dots). The tagging site (Maniitsoq) is indicated by a red dot in each panel and the blue dashed line refers to the Arctic circle. In (**b,c**), the color-coded polygons refer to the light regimes based on solar elevation on July 15 and Dec 15 at sunset in Maniitsoq city: day (angle > 0°), twilight (between 0 and −18°) and night (< −18°). Mean daylength, sunrises and sunsets represent July 15 and December 15 at the porpoises’ locations.

During the period from July to December in both 2013 and 2014, 13,492 locations were recorded from the 17 tagged porpoises (10 females and 7 males, Table 1, Fig. 1a, Fig. SI Fig. 1). With a tracking duration ranging from 16 to 156 days, the individuals experienced contrasting light conditions with a daylength varying from > 19 h in July (Fig. 1b) to < 5 h in December (Fig. 1c).

**Table 1:**
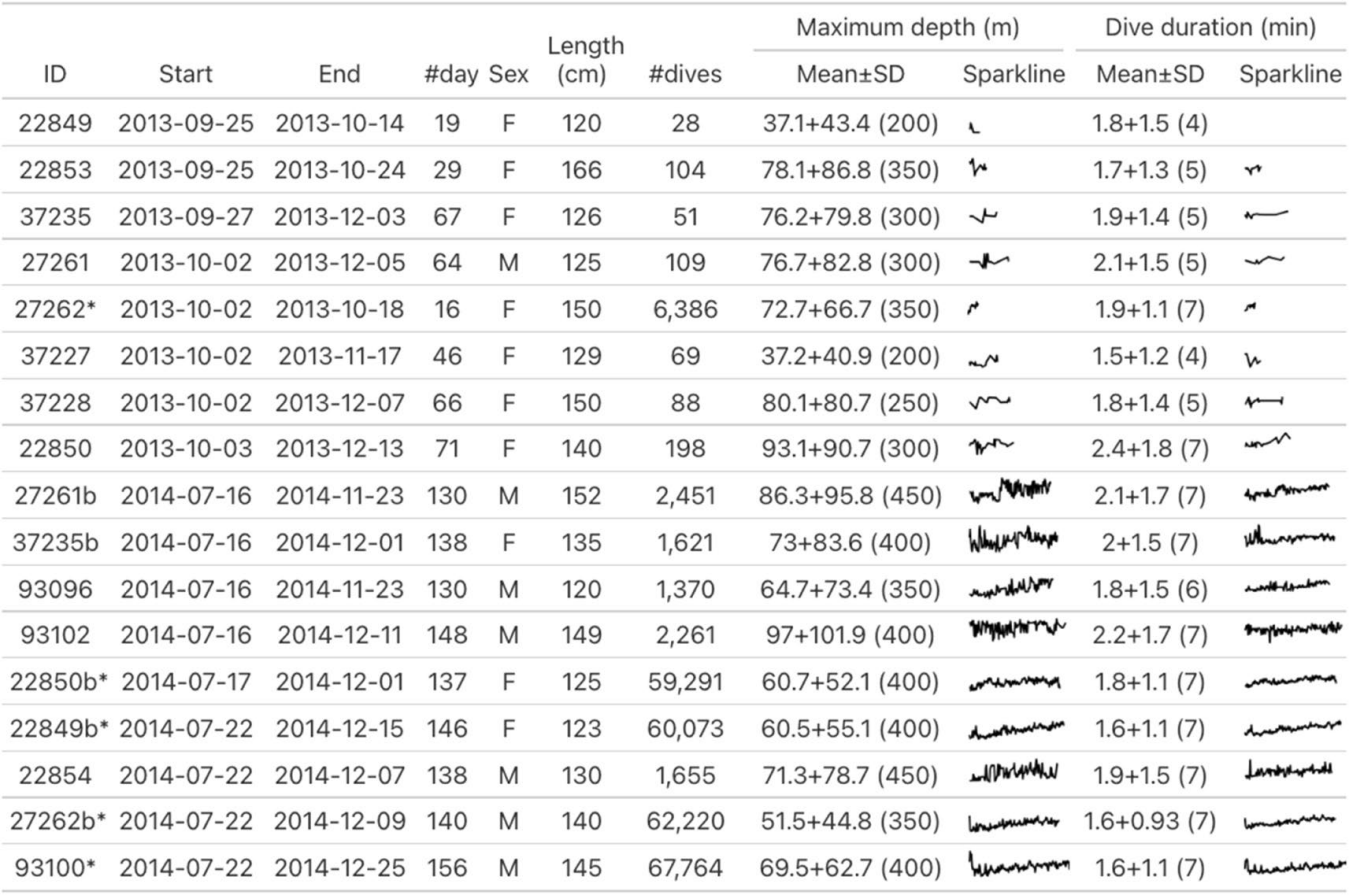
Summary of the dive data for the 17 harbour porpoises instrumented in West Greenland with satellite transmitters.

The sparkline columns show the trend of the variable over time (generally a positive trend in maximum depth (deeper) and dive duration (longer) for each individual during the time of tagging). The numbers in parenthesis refer to the maximum values for each variable, and the individuals with an asterisk (column ID) refer to the tags retrieved and included in the high-resolution data analysis (sampling rate every sec).

### Diel pattern

**Figure 2:**
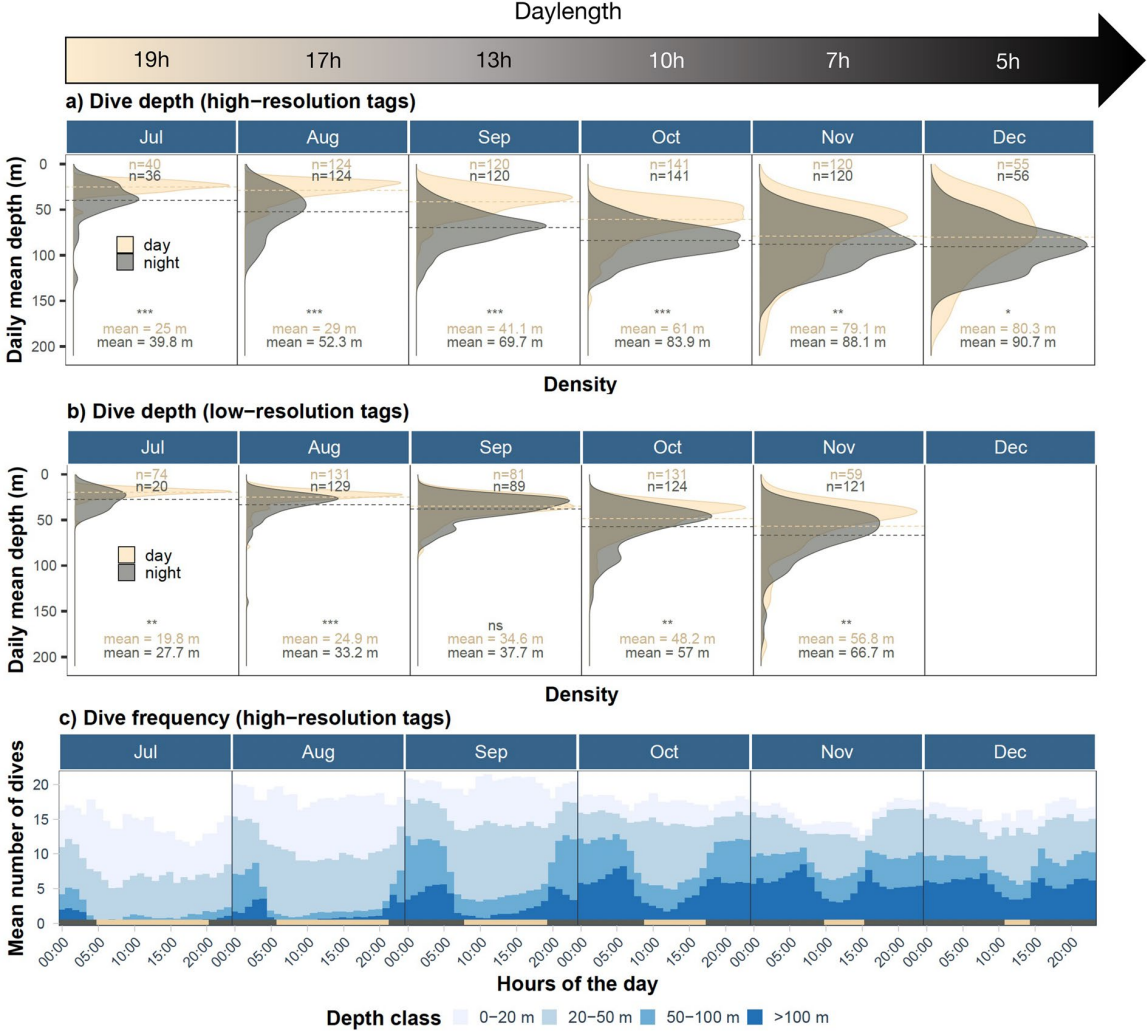
Monthly density plots of the daily mean dive depths recorded by (**a**) the five high-resolution tags and (**b**) the eight low-resolution tags. The monthly mean values for day and night are indicated in each panel and are represented by the horizontal dotted lines. The significance of the associated Kolmogorov–Smirnov tests is indicated in each panel (*ns* not significant, ***p < 0.001, **p < 0.005, *p < 0.05). The number of days for which the mean depth was calculated was added on top of each panel in (**a,b**) for day (beige) and night (grey), respectively. Note that December was discarded from the analysis in (**b**) due to a very low number of dives recorded. (**c**) Stacked histograms of the dive frequency recorded by the five high-resolution tags calculated as the mean hourly dive rate per depth class across months and animals. Note the day/night coloration at the bottom of each panel in (**c**). Average monthly daylength (hours of daylight) was added on the top of each month.

Upon analyzing 265,739 dives, it was found that all the tagged harbour porpoises had a mean daily dive depth of 64 m (SD: 23 m), and most individuals were frequently diving deeper than 100 m (Table 1, Fig. 2). Among the 17 tags, five were retrieved, providing high-resolution data on the diving behavior at a sampling rate of 1 Hz, totaling 255,734 dives. Surprisingly, a reverse diel pattern was observed in the daily mean dive depth for each month as the porpoises dove 37% deeper at night compared to day (mean: 73 m vs. 53 m, Fig. 2a). The Kolmogorov–Smirnov tests were significant for all months, confirming that the distributions of the diurnal and nocturnal dives were statistically different for these five animals. The eight low-resolution tags showed a similar pattern with nocturnal dives being 24% deeper at night compared to diurnal dives (mean: 48.3 m vs. 36.5 m, Fig. 2b). The Kolmogorov–Smirnov tests were significant for all months except September, confirming that the distributions of the diurnal and nocturnal dives were statistically different for these eight animals.

### Seasonal pattern

In addition to the diel pattern, dive depth varied significantly across months (Kruskal–Wallis test, *p* < 0.001) with a pronounced seasonal pattern towards deeper dives when approaching winter months with longer periods of darkness (Fig. 2a, b). As an indication of increased activity, frequency of deep dives (> 100 m) increased by an order of magnitude when daylength decreased (Fig. 2c). When considering dives deeper than 5 m, the porpoises performed on average 17 ± 6.7 dives/h. While the proportion of shallow dives (< 20 m) decreased from July (mean: 7 dives/h) to December (mean: 2 dives/h), the number of hourly dives deeper than 100 m increased substantially between summer and winter (July: 0.4 vs. Dec: 5.3 dives/h), and was concentrated during the dark hours.

### Influence of daylength on the diving behavior

**Figure 3:**
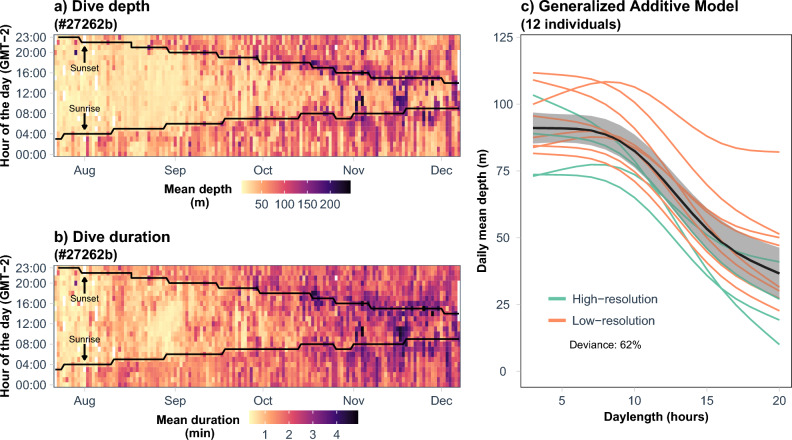
Example of mean dive depth and dive duration showing seasonal and diel patterns for one high-resolution tag deployed in 2014. Black lines in (**a,b**) refer to the hours of sunrise and sunset for the given day. (**c**) Relationship between the daily mean dive depth and daylength for four high-resolution and eight low-resolution tags. In (**c**), the thick line refers to the smooth curve from the GAM including the 12 individuals (with tracking duration > 30 days) and the thin lines depict individual smooth curves.

When looking at the entire study period, shallow (< 50 m) and short dives (< 1 min) occurred mainly during daytime (Fig. 3a, b). The daily mean dive depth was negatively correlated with daylength (r^2^ = 0.61, GAM explained deviance: 62%), revealing that the deepest dives were performed during the darkest months close to the polar night (Fig. 3c). Despite some inter-individual variability, our model revealed a similar behavior across all tracked porpoises, regardless the dataset (high vs. low-resolution tags).

### Influence of the environment on the diving behavior

Overall, the difference between the bathymetry and the maximum dive depth of the porpoises’ indicated that they conducted pelagic (mean ± SD: 47 ± 11% of the dives) and benthic dives (mean ± SD: 45 ± 8%) in similar proportions (SI Figs. 2–6).

**Figure 4:**
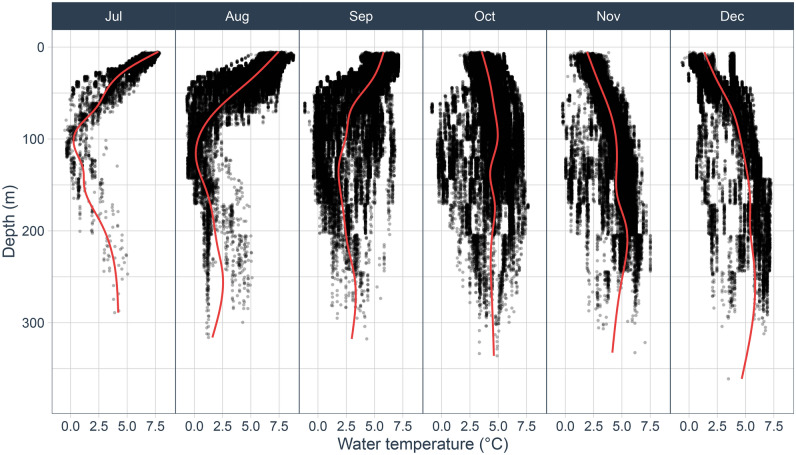
Temperature profiles across months extracted at the closest depth of the porpoises’ maximum dive depth for each location from the five high-resolution tags.

The mean sea temperature at the porpoises’ dive depth was 4.3 ± 0.8 °C and varied between −1.1 and 8.7 °C (Fig. 4). While from July to September, the temperature decreased from the surface to a depth of 50–100 m, a reversed thermocline was observed from October to December, with a more stable temperature, slightly increasing with depth.

## Discussion

The extreme light regimes experienced by the porpoises in Greenland coincided with a clear modulation of the diving activity in response to the seasonal reduction of daylength at high latitude, highlighting an unexpected reverse diel vertical migration. By driving both primary production and the interaction between visual predators and their prey, light has indeed a fundamental influence on the structure of marine ecosystems^28^. Such an interaction, however, is more complex for air-breathing and echolocating predators such as toothed whales than for ectothermic visual predators (e.g., fish and cephalopods) that may be more influenced by thermoregulation and their ability to detect their prey. Indeed, foraging strategies of odontocetes, including harbour porpoises, may be more shaped by prey accessibility, diving constraints and energy conservation. The porpoises maintained their activity at relatively cold temperature (4.3 °C, all depths combined), that likely requires a high feeding activity to sustain a high energetic intake.

The dive depth of a large number of marine mammal species has been identified to be closely associated with the vertical migration of their prey, performing shallower dives at night when the prey ascends towards the surface. Such a pattern has been extensively observed in seals (e.g., Weddell seals^29,30^, fur seals^31^), baleen whales^32^ and toothed whales (e.g., sperm whales^33^, rough-toothed dolphins^12^, Risso’s dolphins^34^), but the association with daylength is to date poorly documented at high latitudes, especially in cetaceans^26^. Unlike most marine predators, our study reports that harbour porpoises conducted a reverse diel vertical migration by diving deeper at night and targeting increasing depths during the fall. This surprising behavior suggests three foraging strategies in which harbour porpoises would either target (i) strictly benthic prey, (ii) pelagic prey migrating seasonally towards the seafloor during fall/winter, or (ii) vertically migrating prey that may be otherwise inaccessible in deeper waters at night. This last strategy suggests that the porpoises could therefore maximize feeding activity during extended periods of darkness. This phenomenon is increasingly more pronounced from summer to winter, reinforcing a light-mediated strategy. The investigation of the thermal habitat of the porpoises revealed a decrease in surface temperature across months and a thermocline shift from October (water temperature increasing with depth). This temperature shift coincides with both the change in light regimes and the increasing dive depth of the porpoises, therefore supporting the assumption that this species might follow the vertical distribution of its prey that is inhabiting deeper layers when the water temperature at the surface decreases and the primary production at the surface pauses during the dark months, making the surface less attractable for zooplankton.

The porpoises in Greenland might not forage much during daytime, remaining in shallow waters while concentrating their feeding activity mostly at night in deeper layers. However, unlike other Arctic toothed whales such as narwhals and belugas that can rely on their body reserves for long periods without feeding^35^, harbour porpoises have high energy demand^17^, and may therefore need to feed continuously to survive. Regularly reaching deep waters (> 100 m), the daily dive duration of the porpoises in Greenland rarely exceeded 4 min (less than 3% of the dives, mean: 1.6 ± 1.02 min, maximum: 7 min, Table 1). In other air-breathing divers, dive duration is typically correlated with body size^36^, and dive depth correlates with dive duration, which is in line with our results (Pearson correlation test: 0.84, *p* < 0.001). The diving capacity of harbour porpoises is therefore strongly limited by their small size, and their aerobic dive limit has been estimated to be about 5 min^37^, suggesting that, despite diving deep presumably to forage, the porpoises rarely exceed their aerobic dive limit.

Although deeper nocturnal dives were observed for all months, the diel pattern was stronger for months with a more pronounced day-night cycle (from July to October), which is in accordance with a previous study conducted on beluga whales in the Chukchi and Beaufort Seas^26^. Weddell seals in Antarctica exhibited a similar behavior (shallower dives in early and mid-summer) that reflected shifts in prey distribution rather than in seal diet composition^30^. This observation could also be the case for the porpoises where their prey may move deeper when the water temperature at the surface decreases through winter. The observed seasonal variability in diving behavior suggests either a temporal switch in prey selection or the porpoises’ tracking of the vertical distribution of specific prey that migrates seasonally within the water column. While harbour porpoises are thought to forage opportunistically, previous studies in West Greenland have identified capelin (*Mallotus villosus*) and polar cod (*Boreogadus* *saida*) as their primary prey towards the end of summer and early autumn (September–October)^25^. Both prey species exhibit vertical segregation by age class, with larger mature individuals residing close to the seafloor (for predator avoidance), making them less reachable to the porpoises (e.g., ~ 400 to 500 m deep in the Beaufort Sea). In contrast, immature individuals of both species are found at higher positions in the water column^38,39^, potentially making them more susceptible to predation by the porpoises. Considering that a significant proportion of the dives was benthic (45% of the dives), it suggests that the porpoises could also target prey near the bottom that are strictly benthic and do not perform diel vertical migration, or pelagic prey seasonally migrating towards the bottom during fall and winter including adult capelins and polar cods.

During summer in West Greenland, harbour porpoises also consume a variety of pelagic prey such as squid, krill and Myctophids (also called lanternfish), suggesting a potential seasonal switch in prey selection. During the summer, porpoises may target immature capelin at shallow depths, while as winter approaches, they may shift their focus to prey at greater depths, such as squids and Myctophidae. Myctophidae are small fish that are abundant in the mesopelagic layer (200–1000 m) and possess lipid reserves for overwintering^40^. By targeting such lipid-rich prey, harbour porpoises could meet their high energetic requirements^41^.

To investigate the role of benthic *vs.* pelagic dives in this species, it will be necessary to deploy acoustic recorders to identify prey capture attempts. In line with this, we used the maximum dive depth as an indication of diving activity, therefore assuming that deeper dives corresponded to a higher number of feeding attempts. Although the lack of acoustic recordings in the present study prevented the investigation of the feeding activity in this echolocating species, the exceptional deep dives observed (both pelagic and benthic) provide further evidence that these dives, primarily conducted during dark hours, are likely indicative of foraging rather than resting behavior. Also, evidence from a study using acoustic tags on porpoises revealed resting behavior during shallow diving to about 7 m^42^. Previous studies have demonstrated that harbour porpoises exhibit greater acoustic activity during the night^17–20^, and actually have higher feeding rates (buzzing) during nocturnal dives^17^. To validate this assumption and confirm when harbour porpoises engage in foraging it will be necessary to deploy acoustic data loggers that includes the identification of buzzes (*i.e.,* foraging events) or accelerometers capable of capturing feeding jerks.

## Conclusion

Aligned with the unexpected levels of biological activity observed during the polar night in low and mid-trophic level organisms^8^, our study provides insights into the impact of daylength on the diving behavior of a marine predator inhabiting sub-Arctic and Arctic waters. Using satellite-linked dive recorders of highly mobile harbour porpoises, we have confirmed that this species adjusts its diving patterns in response to the diminishing daylength, engaging surprisingly in deeper and more frequent dives at night, likely indicative of increased foraging activity during dark hours. Assuming harbour porpoises in Greenland feed more intensively at night—similar to what is observed for their conspecific living in Danish waters^17^—our findings suggest that harbour porpoises in Greenland may capitalize on the winter nightscape of the Arctic^15^. Unlike strictly visual predators, echolocators such as harbour porpoises could therefore extend foraging time and take advantage of long periods of darkness in the winter in the Arctic to optimize their diving activity. Our study further underscores the notion that extreme light conditions observed at high latitudes play a critical role in shaping pelagic communities and food webs. These findings provide new insights for exploring the relationship between foraging strategies, metabolic demands, and fitness of Arctic marine predators in a rapidly changing environment, where prey assemblages are anticipated to undergo shifts in response to climate change^43^.

## Methods

### Animal instrumentation

Between 2012 and 2014, 17 harbour porpoises were live-captured on the continental shelf approximately 50 km south-west of Maniitsoq, West Greenland (Fig. 1). Following Nielsen’s et al*.’s* procedure^27^, the individuals were instrumented with MK10 satellite-linked dive recorders providing information on location and diving behavior (Wildlife Computers, Redmond, WA, USA). The sampling rate was 1 Hz (1 s) and data were stored in 6 h summary histograms (01:00–07:00, 07:00–13:00, 13:00–19:00 and 19:00–01:00) that were relayed to the satellite during the following 24 h. For the tags deployed in 2013, the intervals were: 5, 10, 25 and 50 m, then 50 m bins up to 500 m and then > 500 m. For the tags deployed in 2014, the intervals were: 1, 2, 10, 25 and 50 m, then 50 m bins up to 450 m and then > 450 m. Since the low-resolution tags (the ones not retrieved) were stored into 6 h histograms with pre-defined depth bins rather than a precise depth, we used the lower limit of each bin (deepest depth) to associate a potential maximum depth to each dive.

All experiments involving animals and their care were conducted in compliance with ARRIVE guidelines (https://www.ncbi.nlm.nih.gov) and were performed in accordance with relevant guidelines and regulations. The study was performed with permission from the Government of Greenland, permit no. 2012-069733, Doc. 1265044.

### Identification of diel patterns

All analyses were conducted using R software version 4.3.2^44^. To investigate diel patterns in the diving behavior, sunlight phases (times of sunrise, dusk, day, dawn and sunset) were extracted at each individual’s location using the *suncalc* package^45^ in R. Five tags that were retrieved by local hunters and included only high-resolution dive data (1 Hz) but no coordinates were stored in these tags. Satellite-relayed Argos coordinates were therefore matched to the high-resolution dive data based on the closest date and time for each individual. To map the day-night regimes in relation to the porpoises’ locations in West Greenland, solar elevation was extracted individually at each location and for the corresponding date. Based on the solar elevation angle (i.e., position of the sun in relation to the horizon), three phases were identified: day (solar elevation > 0°), twilight (between 0° and −18°) and night (< −18°). The complete dataset including the 17 individuals was used for this analysis.

For the five high-resolution tags, diving behavior variables (mean dive depth, maximum dive depth, median dive depth and dive duration) were extracted for each dive and individual and then compared between the phases of the day. During midnight sun in summer, dawn and dusk are not always identifiable due to rapid sunsets and sunrises during long periods of daylight, so only two phases were retained for this analysis to compare diurnal and nocturnal dives: day vs. night (including dusk and dawn).

To investigate the diel and seasonal patterns, daily mean depth was then estimated for each individual and then summarized monthly for day and night separately. The short tracking duration (< 30 days) of five out of the 17 porpoises (one high-resolution tag and four low-resolution tags) precluded the identification of seasonal patterns and they were therefore excluded from the dive data analysis. December was also discarded from the low-resolution dataset due to a very low number of 6 h histograms recorded (< 10).

### Investigation of the relationship between daylength and dive depth

Times of sunrise and sunset were used to derive the daylength for each tracking day and each individual for these 12 (four high-resolution and eight low-resolution tags). Using the *mgcv* package ^46^, a series of Generalized Additive Models (GAM) were performed to relate the daylength to the daily dive depth of these 12 harbour porpoises. Dives were defined as deeper than 5 m and longer than 20 s. Three separate models were tested using three diving behavior metrics summarized daily: mean, median and maximum depth. Pairwise correlations between all covariates were first investigated (SI Fig. 7). To account for the inter-individual variability, individual’s ID was added as a random factor on both the slope and the intercept. Individual response curves were then generated for each harbour porpoise. The residuals were finally inspected using diagnostic plots for each model.

### Link between behavior and the environment

To identify if the porpoises conducted benthic or pelagic dives, the bathymetry was extracted from GEBCO (spatial resolution: ~ 1 km, https://www.gebco.net) and then associated with each location. For each dive, the difference between the bathymetry and the animal’s maximum dive depth was calculated. Following Harcourt et al.*’s* method^47^, the 30 percentile of this difference for the dives exceeding bathymetry was then calculated (value: 70 m) as this approximates 1 standard error of a distribution centered on the 1:1 line representing equivalence of a dive depth and estimated bathymetry at that point. The dives that were > 70 m from the bathymetry were categorized as pelagic dives, the ones within 70 m of the bathymetry as benthic dives, and dives more than 70 m deeper than the bathymetry were considered as unknown.

To assess the influence of temperature on the porpoises’ diving behavior, temperature data were extracted daily from the Copernicus Marine Service Information (https://marine.copernicus.eu/) using the GLOBAL_MULTIYEAR_PHY_001_030 product at a resolution of 0.08 decimal degree. For each dive of the five high-resolution tags, the closest temperature to the maximum dive depth was extracted at the animal’s location. The temperature profiles were then plotted monthly to investigate the thermal habitat targeted by the porpoises in relation to the temporal evolution of the thermocline.

### Supplementary Information

Supplementary Figures.

## Data Availability

All the code used for analysis and visualization in this study is available via GitHub (https://github.com/pchambault/HP-Diel-Seasonal-Pattern). The associated data can be found on the Dryad repository (https://datadryad.org/stash/dataset/doi:10.5061/dryad.zpc866tdh).

## References

[1] Daan, S. Adaptive daily strategies in behavior. In *Biological Rhythms* (ed. Aschoff, J.). 275–298 10.1007/978-1-4615-6552-9_15 (Springer, 1981).

[2] Bennie JJ, Duffy JP, Inger R, Gaston KJ (2014). Biogeography of time partitioning in mammals. Proc. Natl. Acad. Sci..

[3] Urmy SS, Benoit-Bird KJ (2021). Fear dynamically structures the ocean’s pelagic zone. Curr. Biol..

[4] Ringelberg, J. *Diel Vertical Migration of Zooplankton in Lakes and Oceans: Causal Explanations and Adaptive Significances*. (Springer, 2009).

[5] Hays, G. C. A review of the adaptive significance and ecosystem consequences of zooplankton diel vertical migrations. In *Migrations and Dispersal of Marine Organisms* (eds. Jones, M. B. *et al*.) 163–170 10.1007/978-94-017-2276-6_18 (Springer Netherlands, 2003).

[6] Fischer J, Visbeck M (1993). Seasonal variation of the daily zooplankton migration in the Greenland sea. Deep Sea Res. Part I.

[7] Berge J (2009). Diel vertical migration of Arctic zooplankton during the polar night. Biol. Lett..

[8] Berge J (2015). Unexpected levels of biological activity during the polar night offer new perspectives on a warming Arctic. Curr. Biol..

[9] Andrzejaczek S (2021). Reverse diel vertical movements of oceanic manta rays off the northern coast of Peru and implications for conservation. Ecol. Solut. Evid..

[10] Sims DW, Southall EJ, Tarling GA, Metcalfe JD (2005). Habitat-specific normal and reverse diel vertical migration in the plankton-feeding basking shark. J. Anim. Ecol..

[11] McIntyre T, Bornemann H, Plötz J, Tosh CA, Bester MN (2011). Water column use and forage strategies of female southern elephant seals from Marion Island. Mar. Biol..

[12] Shaff JF, Baird RW (2021). Diel and lunar variation in diving behavior of rough-toothed dolphins (*Steno bredanensis*) off Kauaʻi, Hawaiʻi. Mar. Mamm. Sci..

[13] Arranz P (2019). Diving behavior and fine-scale kinematics of free-ranging Risso’s dolphins foraging in shallow and deep-water habitats. Front. Ecol. Evolut..

[14] Lampert W (1989). The adaptive significance of diel vertical migration of zooplankton. Funct. Ecol..

[15] Benoit D, Simard Y, Gagné J, Geoffroy M, Fortier L (2010). From polar night to midnight sun: Photoperiod, seal predation, and the diel vertical migrations of polar cod (*Boreogadus saida*) under landfast ice in the Arctic Ocean. Polar Biol..

[16] Donovan, G. & Bjørge, A. *Harbour Porpoises in the North Atlantic: Edited Extract from the Report of the IWC Scientific Committee*. Vol. 16. 3–26 (1995).

[17] Wisniewska DM (2016). Ultra-high foraging rates of harbor porpoises make them vulnerable to anthropogenic disturbance. Curr. Biol..

[18] Osiecka AN, Jones O, Wahlberg M (2020). The diel pattern in harbour porpoise clicking behaviour is not a response to prey activity. Sci. Rep..

[19] Schaffeld T (2016). Diel and seasonal patterns in acoustic presence and foraging behaviour of free-ranging harbour porpoises. Mar. Ecol. Prog. Ser..

[20] Carlström J (2005). Diel variation in echolocation behavior of wild harbor porpoises. Mar. Mamm. Sci..

[21] Linnenschmidt M, Teilmann J, Akamatsu T, Dietz R, Miller LA (2013). Biosonar, dive, and foraging activity of satellite tracked harbor porpoises (*Phocoena phocoena*). Mar. Mamm. Sci..

[22] Wisniewska DM (2018). High rates of vessel noise disrupt foraging in wild harbour porpoises (*Phocoena phocoena*). Proc. R. Soc. B Biol. Sci..

[23] Williamson LD (2022). Spatiotemporal variation in harbor porpoise distribution and foraging across a landscape of fear. Mar. Mamm. Sci..

[24] Nielsen NH, Teilmann J, Heide-Jørgensen MP (2019). Indications of mesopelagic foraging by a small odontocete. Mar. Biol..

[25] Heide-Jørgensen MP (2011). Harbour porpoises respond to climate change. Ecol. Evolut..

[26] Storrie L (2022). Empirically testing the influence of light regime on diel activity patterns in a marine predator reveals complex interacting factors shaping behaviour. Funct. Ecol..

[27] Nielsen NH (2018). Oceanic movements, site fidelity and deep diving in harbour porpoises from Greenland show limited similarities to animals from the North Sea. Mar. Ecol. Prog. Ser..

[28] Kaartvedt S (2008). Photoperiod may constrain the effect of global warming in arctic marine systems. J. Plankton Res..

[29] Heerah K (2013). Ecology of Weddell seals during winter: Influence of environmental parameters on their foraging behaviour. Deep Sea Res. Part II Top. Stud. Oceanogr..

[30] Beltran RS (2021). Seasonal resource pulses and the foraging depth of a Southern Ocean top predator. Proc. R. Soc. B Biol. Sci..

[31] Botha JA (2020). Geographic variation in at-sea movements, habitat use and diving behaviour of female Cape fur seals. Mar. Ecol. Prog. Ser..

[32] Vogel E (2023). Foraging movements of humpback whales relate to the lateral and vertical distribution of capelin in the Barents Sea. Front. Mar. Sci..

[33] Chambault P, Fossette S, Heide-Jørgensen MP, Jouannet D, Vély M (2021). Predicting seasonal movements and distribution of the sperm whale using machine learning algorithms. Ecol. Evolut..

[34] Visser F (2021). Risso’s dolphins perform spin dives to target deep-dwelling prey. R. Soc. Open Sci..

[35] Chambault P, Blackwell SB, Heide-Jørgensen MP (2023). Extremely low seasonal prey capture efficiency in a deep-diving whale, the narwhal. Biol. Lett..

[36] Brischoux F, Bonnet X, Cook TR, Shine R (2008). Allometry of diving capacities: Ectothermy vs. endothermy. J. Evolut. Biol..

[37] McDonald BI (2021). High heart rates in hunting harbour porpoises. Proc. R. Soc. B Biol. Sci..

[38] Luka, G. & Ponomarenko, V. *Seasonal and Daily Vertical Migrations and Structure of Capelin Concentrations in the Barents Sea* (1983).

[39] Geoffroy, M. & Priou, P. Fish ecology during the polar night. In *Polar Night Marine Ecology: Life and Light in the Dead of Night* (eds. Berge, J., Johnsen, G. & Cohen, J. H.). 181–216 10.1007/978-3-030-33208-2_7 (Springer, 2020).

[40] Falk-Petersen I-B, Falk-Petersen S, Sargent JR (1986). Nature, origin and possible roles of lipid deposits in *Maurolicus muelleri* (Gmelin) and Benthosema glaciale (Reinhart) from Ullsfjorden, northern Norway. Polar Biol..

[41] Rojano-Doñate L (2018). High field metabolic rates of wild harbour porpoises. J. Exp. Biol..

[42] Wright AJ (2017). Silent porpoise: Potential sleeping behaviour identified in wild harbour porpoises. Anim. Behav..

[43] Florko KRN (2021). Predicting how climate change threatens the prey base of Arctic marine predators. Ecol. Lett..

[44] R Core Team. *R: A Language and Environment for Statistical Computing*. http://www.R-project.org/ (R Foundation for Statistical Computing, 2021).

[45] Thieurmel, B., Elmarhraoui, A. & Thieurmel, M. *Package ‘Suncalc’*. https://cran.r-project.org/web/packages/suncalc/suncalc.pdf (2019).

[46] Wood, S. N. *Generalized Additive Models: An Introduction with R*. 2nd edn. (CRC Press, 2017).

[47] Harcourt R (2021). Regional variation in winter foraging strategies by Weddell seals in eastern Antarctica and the Ross Sea. Front. Mar. Sci..

