# Supplementary material for: The nightscape of the Arctic winter shapes the diving behavior of a marine predator
Source: Sci Rep. 2024 Feb 16;14:3908. doi: 10.1038/s41598-024-53953-w (PMC10873309; doi:10.1038/s41598-024-53953-w)
Supplement: Supplementary file 1 — Supplementary Figures. [file 41598_2024_53953_MOESM1_ESM.pdf]

SUPPLEMENTARY INFORMATION for:

The nightscape of the Arctic winter shapes the diving behavior of a marine predator

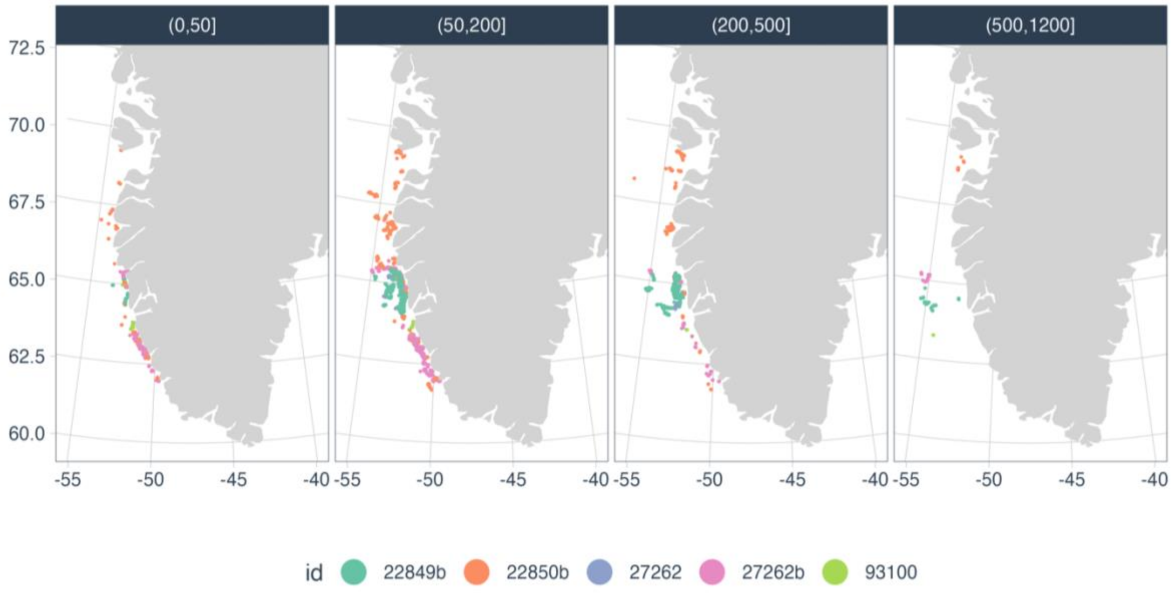

SI Figure 1. Locations of the five high resolution tags according to four bathymetry classes (in m) extracted at the porpoises locations.

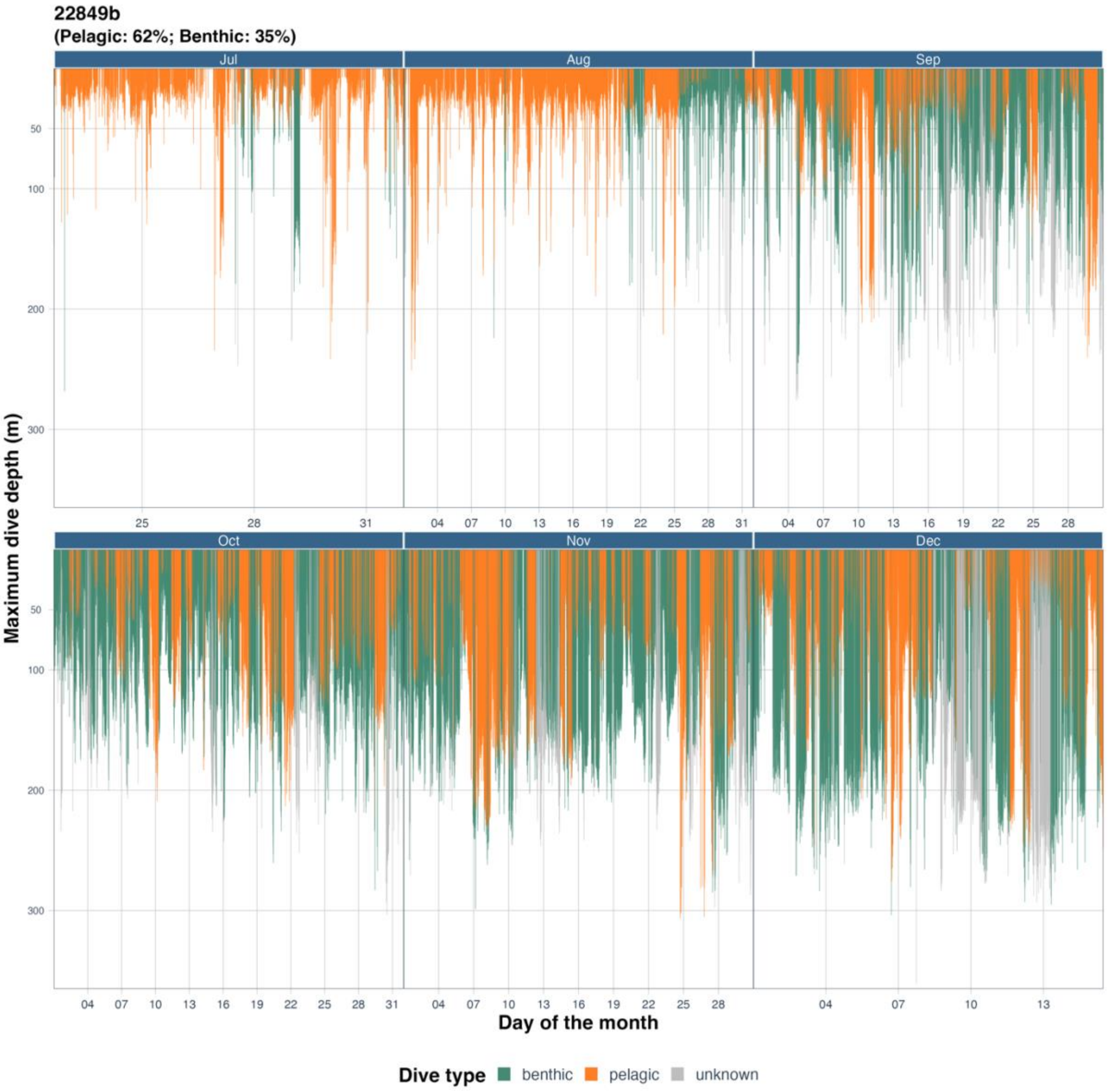

SI Figure 2. Dive profile of the individual 22849b showing the maximum dive depth according to the dive type (benthic in green vs. pelagic in orange).

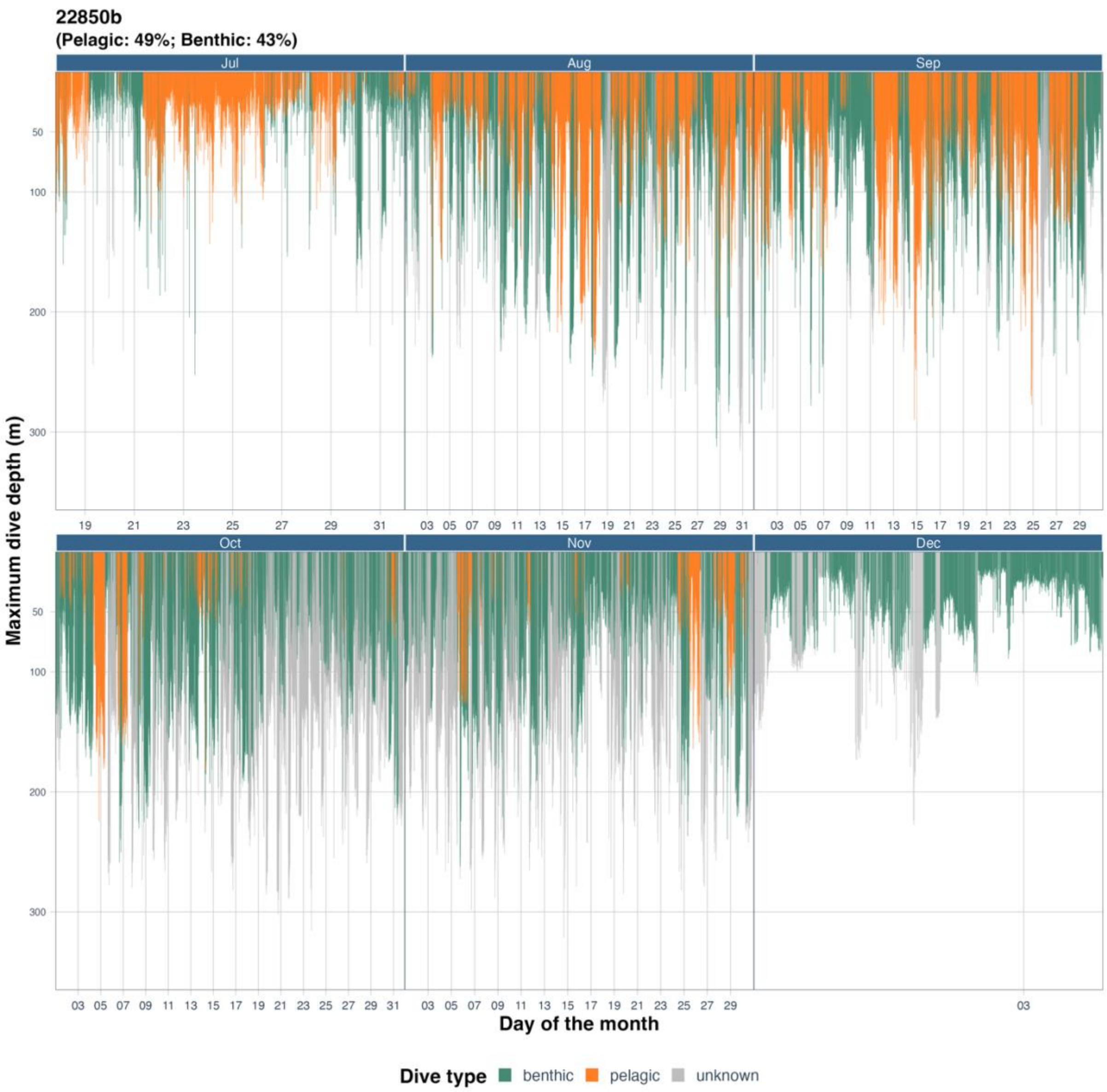

SI Figure 3. Dive profile of the individual 22850b showing the maximum dive depth according to the dive type (benthic in green vs. pelagic in orange).

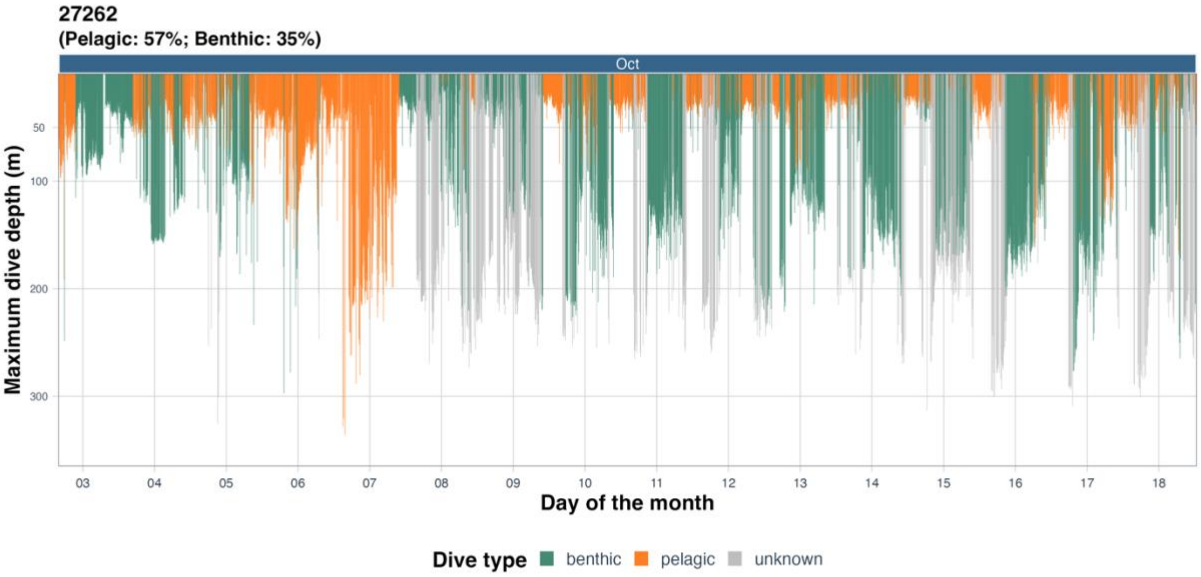

SI Figure 4. Dive profile of the individual 27262 showing the maximum dive depth according to the dive type (benthic in green vs. pelagic in orange).

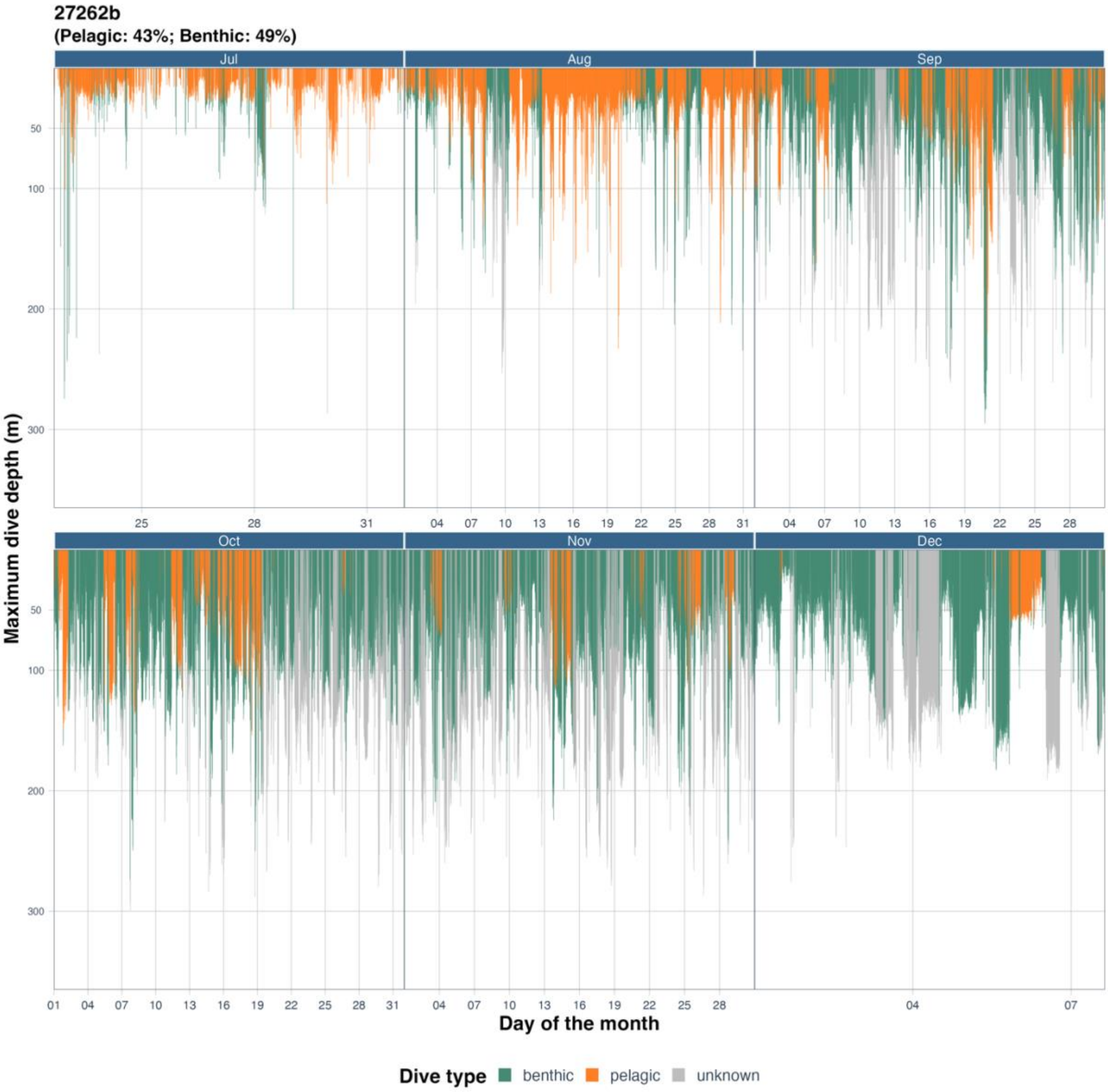

SI Figure 5. Dive profile of the individual 27262b showing the maximum dive depth according to the dive type (benthic in green vs. pelagic in orange).

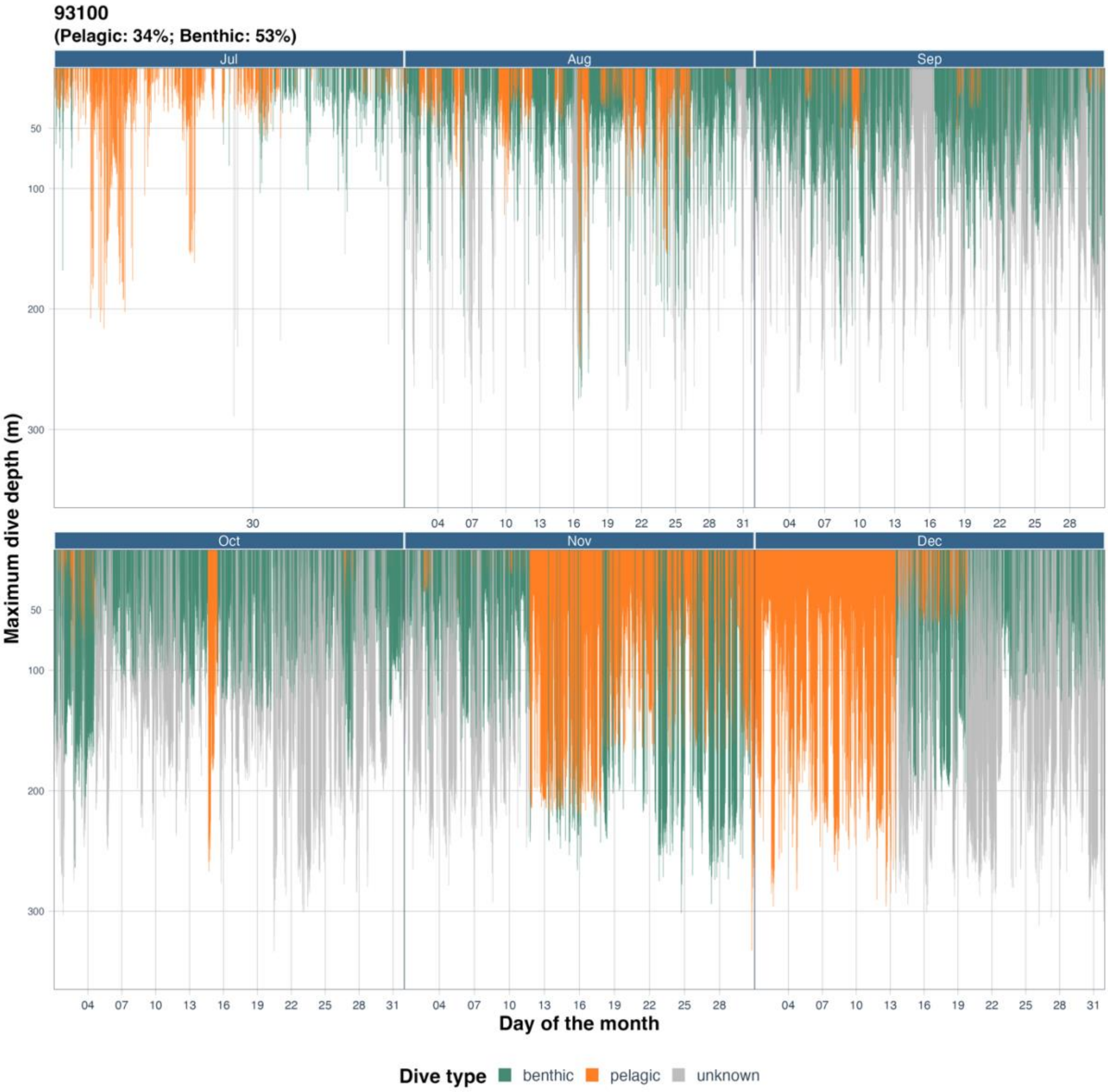

SI Figure 6. Dive profile of the individual 93100 showing the maximum dive depth according to the dive type (benthic in green vs. pelagic in orange).

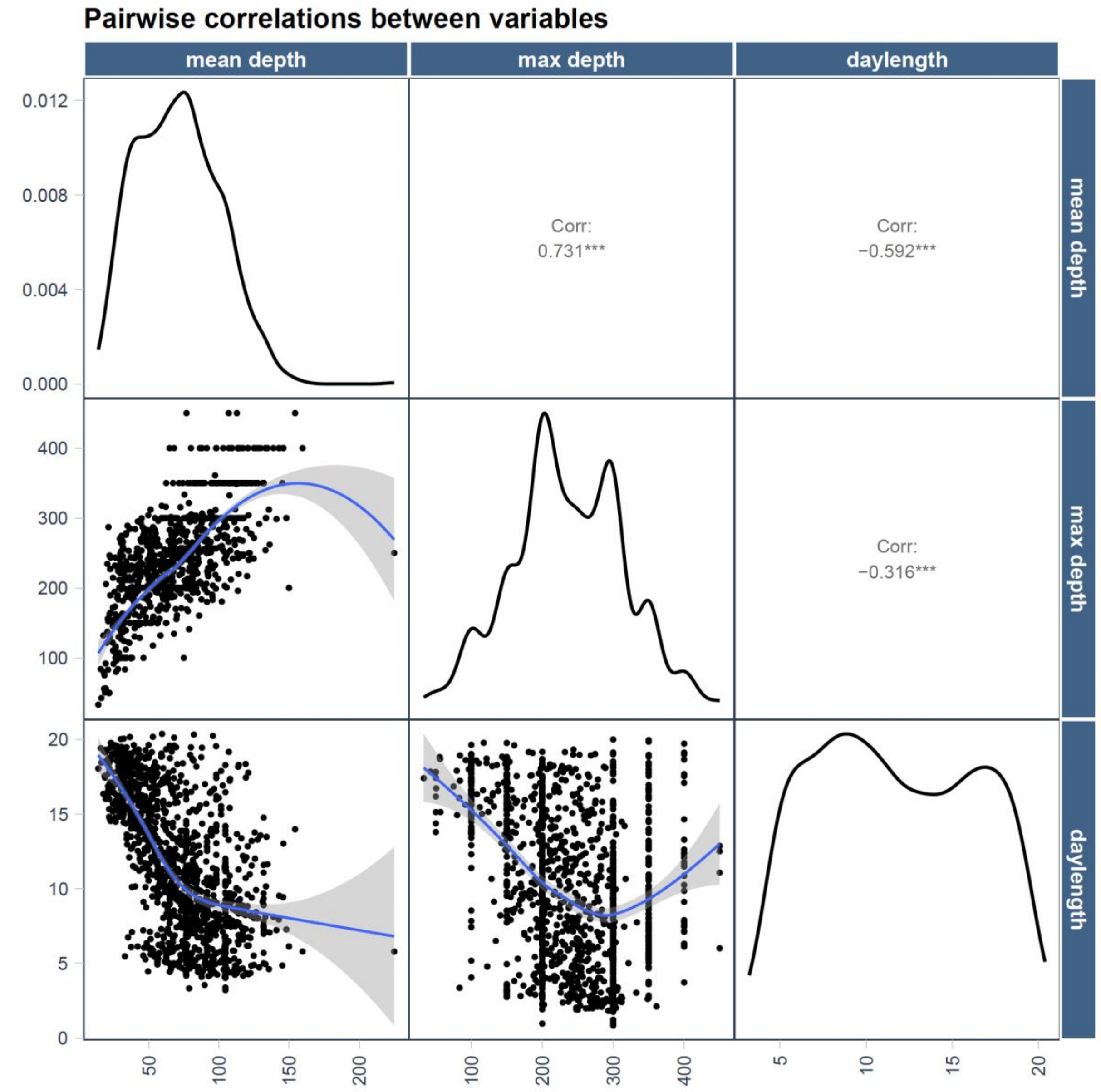

SI Figure 7. Scatterplots showing the pairwise correlations between each covariate. The distribution and correlation coefficients are also shown in each panel.
